# Developing a Service Platform Definition to Promote Evidence-Based Planning and Funding of the Mental Health Service System

**DOI:** 10.3390/ijerph111212261

**Published:** 2014-11-26

**Authors:** Yong Yi Lee, Carla S. Meurk, Meredith G. Harris, Sandra Diminic, Roman W. Scheurer, Harvey A. Whiteford

**Affiliations:** 1 School of Population Health, University of Queensland, via Level 2, Public Health Building, Herston Road, Herston, QLD 4006, Australia; E-Mails: c.meurk@qcmhr.uq.edu.au (C.S.M.); meredith_harris@qcmhr.uq.edu.au (M.G.H.); sandra_diminic@qcmhr.uq.edu.au (S.D.); h.whiteford@sph.uq.edu.au (H.A.W.); 2 Policy and Epidemiology Group, Queensland Centre for Mental Health Research, Queensland Health, via Queensland Centre for Mental Health Research, The Park Centre for Mental Health, Locked Bag 500, Sumner Park BC, QLD 4074, Australia; E-Mail: roman_scheurer@qcmhr.uq.edu.au

**Keywords:** service platform, health systems, mental health, priority setting, decision making, health planning, health policy, cost-effectiveness, Delphi method, qualitative analysis

## Abstract

Ensuring that a mental health system provides ‘value for money’ requires policy makers to allocate resources to the most cost-effective interventions. Organizing cost-effective interventions into a service delivery framework will require a concept that can guide the mapping of evidence regarding disorder-level interventions to aggregations of services that are meaningful for policy makers. The ‘service platform’ is an emerging concept that could be used to this end, however no explicit definition currently exists in the literature. The aim of this study was to develop a service platform definition that is consistent with how policy makers conceptualize the major elements of the mental health service system and to test the validity and utility of this definition through consultation with mental health policy makers. We derived a provisional definition informed by existing literature and consultation with experienced mental health researchers. Using a modified Delphi method, we obtained feedback from nine Australian policy makers. Respondents provided written answers to a questionnaire eliciting their views on the acceptability, comprehensibility and usefulness of a service platform definition which was subject to qualitative analysis. Overall, respondents understood the definition and found it both acceptable and useful, subject to certain conditions. They also provided suggestions for its improvement. Our findings suggest that the service platform concept could be a useful way of aggregating mental health services as a means for presenting priority setting evidence to policy makers in mental health. However, further development and testing of the concept is required.

## 1. Introduction

Mental disorders are the leading cause of health related disability worldwide [1]. A study undertaken for the World Economic Forum estimates that the cumulative global impact of mental disorders in terms of lost economic output may amount to US$16 trillion over 20 years, equivalent to 25% of global GDP in 2010 [2]. Despite the extent of disability, personal suffering and economic costs on individuals and their families, treatment rates for people with mental disorders remains low with an average treatment gap of 50% in all countries of the world, which is up to 90% in some impoverished nations [3,4]. While a lack of human and financial resources to provide effective interventions is often cited as the cause of low treatment rates, inequities in the distribution of available resources and inefficiencies in their use are also major impediments [5]. 

Mental health reform is a complex and cyclical process [6]. In Australia, as in many countries, reform has historically been an incremental process, more often than not initiated by perceived crises, sentinel events or lobbying by policy advocates [7,8]. This piecemeal approach to health system reform—and the complex funding arrangements between governments, providers and service users—has contributed to a fragmented, less efficient system that averts less burden than would be possible with an efficient allocation, for a given level of expenditure [8,9]. Evidence-based priority setting is an important factor for the establishment of fair and efficient mental health systems globally [10,11]. Improving the efficiency and equity of the mental health system requires a fair and effective means of allocating resources across the range of interventions that can cost-effectively reduce the burden of mental disorders [12,13].

The mental health system is complex and confounded by numerous issues, such as: the multifaceted needs of patients (esp. people with severe and persistent mental illness); irregularities in the quality of service provision; the unpredictably of treatment outcomes; the variability in clinician skill and experience; the multiplicity of possible treatments for some conditions; and diverse treatment-seeking behaviors and preferences in the population [3,14]. Allocating resources to evidence-based interventions will not in and of itself produce an efficient and equitable mental health system. Such allocations nonetheless provide an important and necessary step towards effective population-based mental health planning.

In Australia, as elsewhere, there have been endeavors to expand the evidence base for mental health priority setting [15,16,17,18]. Previous studies have produced robust evidence on the cost-effectiveness of disorder-level interventions to decrease the mental health burden in Australia [17,18]. Likewise, service models describing optimal mental health systems have also been developed [15,16]. However, a systematic approach incorporating the best available priority setting evidence has never been used to strategically re-design Australia’s mental health system [8]. It has been suggested that this, in part, reflects a mismatch between the way conventional cost-effectiveness evidence is produced and the way mental health policy makers plan and fund services [8]. For instance, health service planners are regularly tasked with allocating resources across aggregated entities of the mental health system (e.g., primary care or bed-based hospital services). On the other hand, researchers (e.g., clinicians and health economists) conventionally present evidence according to the types of interventions that should be funded to address individual disorders (e.g., selective serotonin reuptake inhibitors for depression or internet-based cognitive behavioral therapy for anxiety). 

It follows, then, that policy makers could potentially be more receptive to evidence presented in terms of meaningfully aggregated service system entities, rather than disorder-level interventions [8]. For example, the burden able to be averted in primary care compared to inpatient services might be more relevant for policy makers than whether pharmacotherapy is more cost effective than a particular type of psychological therapy. Organizing cost-effective interventions into a service delivery framework will require the development of a concept that can guide the mapping of evidence on disorder-level interventions to aggregations of services that are meaningful for policy makers [8].

The ‘service platform’ is a concept that has recently gained traction in the health economic literature [19,20,21,22], although no precise definition exists. The concept is currently being used by the Disease Control Priorities Project (DCPP) to promote evidence-based priority setting in low- and middle-income countries. In this context, platforms describe elements of the service system such as community health clinics, hospitals, schools and public health services that form the basis for the economic evaluation of health care initiatives [19]. Moreover, the ‘service platforms’ developed by DCPP have limited generalizability to developed country settings as mental health systems in high-income countries, such as Australia, are generally characterized by greater breadth and complexity.

We propose that a definition of service platforms is needed that meaningfully classifies the different categories of services in a mental health system. It should also reflect how services are pragmatically grouped for the purposes of planning and funding. A definition such as this could be the foundation for constructing a taxonomy that comprehensively describes a mental health system. An approach to planning centered on platforms could thus provide guidance on how to systematically restructure a mental health system and will complement previous models for rational mental health planning, such as the ‘mental health matrix’ by Thornicroft and Tansella [23] and the ‘European Service Mapping Schedule’ [24]. It should be noted that the service platform concept intends to classify elements of the mental health service system independently of population health needs. The rationale for this is that any given platform may be suitable for delivering services to multiple need groups and that the nature of those groups may vary over time (e.g., in response to changes in evidence). It follows that the platform concept should have flexibility to account for these evolving need groups.

This paper documents the first stage of our engagement with policy-makers to develop a concept and taxonomy of service platforms. Based on our understanding of the policy making process, we expected that a latent conception of service platforms exists among policy makers in the mental health sector. However, the lack of a definition hinders the further use of this concept in service system planning and priority setting. Researchers and the end-users of research (for our purposes, policy makers) need to jointly develop the service platform concept so that it can feasibly influence the decision making process [13]. This study aimed to achieve this by: (i) developing a service platform definition that is consistent with how policy makers conceptualize the major elements of the mental health service system; and (ii) testing the validity and utility of this definition through consultation with a panel of mental health policy makers.

## 2. Methods

### 2.1. Developing a Definition of a Service Platform 

A provisional definition of service platforms was developed through an iterative process involving both a review of the published literature and expert feedback from a group of researchers who are part of the Australian Centre for Research Excellence in Mental Health Systems Improvement (CREMSI) [25]. We first conducted a structured literature search to examine how service platform terminology has been used in the health literature. Because ‘service platform’ is a sufficiently generic phrase, with numerous context-specific meanings, we judged that a systematic literature review would be an impractical and unhelpful approach to the task of generating a useful definition. That is, we anticipated that a search for a particular instance of the term would become intractable given its multiple usages. Nonetheless, we thought it worthwhile to conduct a crude search of the term to determine the ways in which it is broadly applied. An unrestricted search of the terms ‘service platform’, ‘health service delivery platform’ and other variants was performed in PubMed on June 2013.

Our search located 125 relevant studies. An examination of abstracts revealed that the term ‘service platform’ was used in four different ways to denote: (i) an element or entity of a health service delivery system; (ii) an information technology platform for the delivery of e-health or health-related mobile phone services; (iii) health care interventions that serve as a gateway for the delivery of further interventions; or (iv) a political platform by which political candidates espouse their declared principles or aims in relation to health policy. The first classification best fit our sense of what a service platform is for the purposes of planning. However, the search failed to locate an explicit definition of such platforms. The absence of an existing definition led us to formulate our own.

The development of the service platform definition was guided by several existing models from the mental health planning literature. Accepting that there is a mental health intervention spectrum [26,27,28,29], we developed a definition that could be used to delineate discrete platforms according to an intended function and which, altogether, covered the intervention spectrum for mental disorders—*i.e.*, promotion, prevention, early intervention, treatment and continuing care (or recovery). 

The Health Resource Group (HRG) classification system influenced the service platform definition [30,31,32]. HRGs are defined as ‘groupings of interventions that are similar in resource type and organization, including the setting of care’ [32]. HRG’s have been used in a previous study to classify health care provision and associated costs according to five general groups that were matched to population health needs—*i.e.*, health promotion, prevention, clinical primary health care (new cases), clinical primary health care (existing cases) and hospital care [30].

The National Mental Health Service Planning Framework (NMHSPF) recently constructed an Australian population-based planning model, which matches service elements and care packages to the needs of the population. The idea of a service element was an antecedent to the service platform concept [33]. The NMHSPF is a significant step towards designing an optimal mental health service system.

Two previous pieces of research have sought to facilitate rational mental health planning, with each developing a conceptual framework to systematically describe components of a mental health system. The first is the ‘mental health matrix’ by Thornicroft and Tansella [23] which is composed of two dimensions: (1) the ‘geographical’ dimension which comprises three levels—*i.e.*, a country/regional, local and individual level; and (2) the ‘temporal’ dimension which comprises three phases—*i.e.*, an input, process and outcome phase. Mental health strategies and sentinel events can be understood in a broader planning context by simultaneously mapping relevant attributes across these two dimensions of space and time in a 3 × 3 matrix. The second is the ‘European Service Mapping Schedule’ which enables the classification of mental health services within defined geographical catchment areas according to a set of organizing principles [24]. These principles include: constructing a taxonomy of services that could fit the major service models used in Europe; deriving operational definitions of services based on their function, with a focus on what is being delivered; having content validity to clinicians and service planners; and being useable as the basis for quantitative assessment of service provision in a catchment area.

Information from the aforementioned literature was used to inform the development of a service platform definition. Expert feedback was then elicited from a team of Australian researchers with extensive experience in the fields of mental health policy, health economics and service system planning [25]. The definition was amended in response to this feedback.

### 2.2. Testing the Validity of the Definition 

#### 2.2.1. Recruiting a Sample of Mental Health Policy Makers

We chose to elicit feedback from a purposive sample of policy makers who play an active role in executive-level decision making and the formulation of mental health policy in Australia. We viewed active collaboration with the end-users of this research, policy makers in this instance, as part of an ongoing, bidirectional, knowledge sharing process. We sought to elicit the views of mental health policy makers to ensure that we produce priority setting evidence that is relevant to their needs as decision makers. We also saw this process as part of a wider knowledge transfer process that could facilitate the translation of findings from research into policy. The sample comprised a group of Australian policy makers who agreed to be part of a Policy Advisory Panel (PAP) as part of the Centre of Research Excellence in Mental Health Systems Improvement (CREMSI) [34]. A total of 14 Australian policy makers were invited to join the PAP. The composition of invited participants included: senior bureaucrats from both Federal and State government departments of health; members of the Federal and State mental health commissions; representatives from the private health sector; and members of peak non-government mental health organizations. Prospective participants were chosen to ensure a representative set of policy makers that could provide us with feedback on the decision making context for the typical mental health policy maker in Australia. Each prospective participant was emailed a letter of invitation with an accompanying participant information sheet. Of the 14 invited policy makers, 12 consented to participate in our study by signing a written statement and returning this to the study authors. Ethical approval for the CREMSI PAP was obtained from the University of Queensland (#2014000546).

#### 2.2.2. Procedure for Validating the Proposed Definition

A modified Delphi method was employed to elicit feedback on the acceptability, comprehensibility and usefulness of the service platform concept. The Delphi technique is a structured group communication tool used to elicit the opinions of ‘experts’ through two or more rounds of questionnaires [35,36]. The technique was originally designed as a group communication process that aims to achieve a convergence of opinion on a specific real-world issue [37]. Questionnaires are anonymously completed to prevent the personality or influence of some participants from dominating others in the process [37].

Consenting participants were sent a questionnaire that elicited their views on the service platform definition through a series of six questions (see Table 1). Question 1 directly asked the respondents if they considered the definition to be acceptable. Questions 2 and 3 sought to test whether our definition of a service platform was comprehensible. The former question asked respondents outright if they understood the concept, while the latter asked them to name explicit examples. Questions 4 and 5 asked the respondents to comment on the usefulness of the service platform concept as: (i) a way of conceptualizing the mental health system; and (ii) a tool to facilitate planning and funding decisions. Question 6 asked respondents to share any additional comments. Respondents who did not return a completed questionnaire within one week were sent two follow-up reminders, both a week apart. Of the 12 consenting participants, a total of eight completed and returned the questionnaire. One respondent, who was too busy to provide a written response, provided verbal comments to CM—leading to a total of nine respondents. An original copy of the service platform questionnaire is contained in the supplementary material.

#### 2.2.3. Analysis of Transcripts

We used the following protocol to collect and analyze questionnaire responses: (i) CM was responsible for distributing the questionnaire, following up participants and collecting responses; (ii) RS collated the responses for each question in a de-identified format using the NVivo (Version 10) software; and (iii) YL coded and analyzed de-identified responses with assistance from the other study authors. To remove the likelihood of bias, the person responsible for coding and analyzing the data was blinded to the identities and organizational affiliations of the respondents. We consequently analyzed questionnaire responses in a democratic fashion that did not account for respondent level of experience, power/influence, seniority or institutional perspective (e.g., national *vs.* state, public *vs.* private, *etc*.).

**Table 1:** Service Platform Questionnaire.

One definition of a service platform is: “A service platform is a grouping of related services that are similar in resource type and constitute a component of a continuum of care.”	
Q1.	Can you tell us if this definition of a Service Platform is consistent with how you think the health system is organised? Why or why not?
Q2.	Does this concept make sense to you? Why or why not?
Q3.	Can you provide us with examples of Service Platforms?
Q4.	Do you think this definition of a Service Platform is a useful way to conceptualise the mental health system? Why or why not?
Q5.	Do you think this definition of a Service Platform delineates different types of services in a way that is helpful for planning and funding?
Q6.	Is there anything else about the Service Platform concept that you would like to share with us?

A written prompt for each survey question was prepared in the advent that a respondent had difficulty interpreting a particular question and requested further clarification. The purpose of this was to be systematic and consistent if the study authors were asked to clarify questions through an email or verbal exchange. No respondent requested further clarification.

A particular issue relevant to this study was how to analytically deal with disagreement (or negative responses) towards the service platform definition, given the authors’ positions as stakeholders with respect to this definition. We hypothesized that a negative response could be due to a lack of understanding or misunderstanding of either the definition itself or the question being asked. However, a negative response could also be the result of authentic disagreement with the researchers that stems from the poor acceptability, comprehensibility or usefulness of the definition. In advance of receiving transcripts we devised a protocol on how to deal with negative responses to reduce the likelihood of confirmation bias. In the analysis, we resolved to: (i) interpret and classify comments in terms of agreement or disagreement with the proposed definition; and (ii) assess the level of agreement among respondents themselves and cluster responses accordingly.

Questionnaire responses were initially analyzed using a deductive approach that assessed responses in terms of the aforementioned framework for analysis—*i.e.*, acceptability (Q1), comprehensibility (Q2 and Q3) and usefulness (Q4 and Q5). The study authors established a set of codes to analyze relevant ‘a priori’ responses anticipated for each question (see Table 2). We also searched transcripts to identify any key words or ideas proffered by respondents that could be used to amend the service platform definition. Several important themes related to the decision maker perspective emerged during the course of the analysis. Additional codes were developed by YL to uncover these themes and were revised during regular meetings with other authors. We sought to analyze transcripts to the point of saturation—that is, when no further categories are discovered or constructed based on examination of data [38].

**Table 2:** Service Platform Questionnaire.

Framework of Analysis	Code
Acceptability (Q1)	EXP ACC (explicit acceptance)
	PART ACC (partial acceptance)
	EXP REJ (explicit rejection)
Comprehensibility (Q2)	EXP COMP (explicit comprehension)
	TAC & COND COMP (tacit and conditional comprehension)
	EXP NOT COMP (explicitly does not comprehend)
User generated examples (Q3)	CONV (convergent understanding of service platforms)
	DIV (divergent understanding of service platforms)
Usefulness (Q4–Q5)	EXP USE (explicit usefulness)
	TAC USE (tacit usefulness)
	COND USE (conditional usefulness)
	EXP NOT USE (explicitly states that the definition is not useful)

## 3. Results

### 3.1. Developing a Definition of a Service Platform 

Our preliminary investigations of the literature and discussions with experts led us to develop the following definition of a service platform:

*“A service platform is a grouping of related services that are similar in resource type and constitute a component of a continuum of care.”* 

Examples of possible service platforms include: a ‘primary mental health care’ platform including services provided by general practitioners; a ‘bed-based mental health care’ platform comprising inpatient care and overnight residential care services; and a ‘psychosocial support services’ platform that encompasses support and recovery services.

### 3.2. Testing the Validity of the Definition 

#### 3.2.1. Acceptability of the Service Platform Definition (Q1)

Question 1 tested the acceptability of the service platform definition by directly asking PAP respondents if they thought the definition was consistent with how the mental health system is structured. Responses were classified according to the degree of acceptance expressed by each respondent (*i.e.*, explicit acceptance, partial acceptance, explicit rejection). Participants mostly agreed with the definition with certain caveats. A key issue that emerged was whether the proposed definition reflected the actual system or an ideal system, and the practical consequences of this.

Two respondents overtly accepted the definition. For example, one respondent stated:

*“Yes. In mental health we could think of community based services and bed based services as two service platforms given the current model of funding and comparability of function. […] So the definition is reasonable but may still need to be further broken down depending on the use.”*

*[Respondent 2]* 

Four participants expressed partial acceptance of our definition. This acceptance was qualified in one of two ways. First, were those who agreed the definition had value in an ideal sense for planning and modelling, but may not adequately describe the current system:

*“This is an “ideal” definition for the purposes of model building. The service is not organized in a completely rational manner and is the consequence of historical and societal influences. […] Current services are not usually organized along a continuum of care (although I believe they should be).”*

*[Respondent 8]* 

Secondly, one respondent expressed concern that the definition did not explicitly reference population needs:

*“To some extent, but I worry about there being no mention of the service platform being related to the needs of the service population.”*

*[Respondent 5]* 

Three respondents explicitly rejected the definition. Two were unsure if they correctly understood the definition, while the third echoed the comments of those who partially agreed by highlighting the distinction between the ideal and current health systems:

*“The health system is currently organized largely on source of funding not necessarily on the grouping of related services to be delivered. So in this sense I do not think a ‘Service Platform’ is consistent with the way the system is organized, however, I do believe that if we are to have a reflection of the continuum of care, a service platform approach is a good way of doing it.”*

*[Respondent 6]* 

Overall, we found that the majority of PAP respondents (six) expressed some degree of acceptance towards the service platform definition. However, including the notion of a ‘continuum of care’ in the definition led respondents to question the pragmatics of the concept. They argued that the complex organization of the current system does not reflect a continuum of care. On the other hand, many expressed that the service platform approach would be suitable for mapping an ideal service system. The views of those who explicitly rejected the definition echoed the views of those who agreed, insofar as they stated that the definition is aspirational in nature. There was disagreement among respondents as to whether the service platform definition reflected the current model of funding.

#### 3.2.2. Comprehensibility of the Service Platform Definition (Q2)

Question 2 directly asked PAP respondents if they understood the service platform concept. In deliberating the comprehensibility of the definition, the majority of participants began to actively reflect on the implications and usefulness of the service platform approach. We ordered responses with respect to three categories: explicit comprehension; tacit comprehension; and those who explicitly did not comprehend.

Four people explicitly understood the definition. In one case, the respondent correctly identified the link between the definition we provided and an information source from which our definition was derived (*i.e.*, NMHSPF):

*“This concept does make sense to me. In the development of the National Mental Health Services Planning Framework (NMHSPF) we attempted to categorise the system in a hierarchical taxonomy of Service Group/Stream/Category/Element/Activity/Quantification.” *

*[Respondent 6]* 

In one case, a respondent (*i.e.*, Respondent 5 from the previous section) indicated that they understood the definition but reiterated their criticism that it does not reference the needs of the service population.

Three respondents did not communicate explicit comprehension of the definition, but appeared to convey an implied understanding through their deliberations. All three respondents reflected on how they would use the service platform approach to model a mental health service system. In so doing, they referenced ideas relevant to the service platform concept, albeit in a sometimes critical manner. Participants again considered how the service platform approach might stand in relation to ideas that are not currently included in the definition, but could affect its applicability to a ‘real’ rather than ‘ideal’ system:

*“I believe the definition would be a good starting point for model construction, if it also included the concept of “problem or disorder type” as well as resource type and continuum of care.”*

*[Respondent 8]* 

*“The concept seems to presume the existence of a “continuum of care”, but this is not something that many experience. Many experience fragments of service, largely disconnected from each other. Too often components of a system are considered on their own, rather than as a link in a chain that requires many components if any one component is to be successful.”*

*[Respondent 9]* 

Two people expressed that they did not understand the definition. One of these pointed out the source of their confusion:

*“Not really—I’m confused as to why a ‘grouping’ is only a ‘component’.”*

*[Respondent 1]* 

A complementary response was given during an interview with Respondent 7, who had difficulty understanding the concept. The respondent stated that they were unfamiliar with the term ‘service platforms’ and instead used the terms ‘service clusters’ or ‘service constellations’.

#### 3.2.3. Examples of Service Platforms Provided by Respondents (Q3)

In the previous question, we directly tested respondent comprehension by assessing the self-reported understanding of the definition. The second method for (indirectly) testing comprehension was to ask PAP participants to list specific examples of a service platform. The pattern of responses fell into one of two categories, which we described as ‘convergent understanding’ (*i.e.*, respondent examples corresponded with aggregated service entities with a defined function) and ‘divergent understanding’ (*i.e.*, respondent examples did not correspond with aggregated service entities with a defined function). The examples for eight of the nine respondents were analyzed, as one did not provide an answer.

Seven people (out of eight) provided convergent examples:

*“In the current system […] I think there is a fairly common understanding of related services that are delivered through a community based platform, bed based, outreach etc. Within each there is a different range of service types provided although with some overlap—e.g., medication information and provision, family/psychological interventions. The platforms are accessed at different phases of care and mostly form a continuum.”*

*[Respondent 2]* 

*“In patient care, primary care, psychosocial support”*

*[Respondent 9]* 

Some respondents appeared to augment the idea of a service platform with other factors considered important to service planning, such as the specification of target groups by age or disorder type:

*“Acute Inpatient services grouped older persons, adult, child and youth. Community continuing care teams split older persons, adult, child and youth. Acute care teams general adult. High security inpatient services etc.”*

*[Respondent 3]* 

*“Some examples of Service Platforms might include collaborative care models in primary for depressive disorders, services for neuro-degenerative disorders (Huntington’s Disorder Services in Victoria), Mothers and Babies Services.”*

*[Respondent 8]* 

Respondent 4 named a mental health organization (*i.e.*, headspace) as a service platform:

*“headspace is an example of a service platform that seeks to link consumers through a single point of contact to a range of services.”*

*[Respondent 4]* 

One respondent provided a protocol as an example that was classified as neither divergent nor convergent:

*“A bit tricky because I’m not clear, but my guess is most chronic disease management protocols would look something like this.”*

*[Respondent 1]* 

We found that seven out of nine respondents articulated an explicit or tacit understanding of the service platform concept when assessing self-reported comprehension. Likewise, our indirect test for comprehensibility led to seven out of eight participants naming examples that were in the form of aggregated service system entities with a defined function—albeit at differing levels of aggregation. Several respondents also provided examples that augmented the platform concept with other factors that they may have considered important to mental health planning.

#### 3.2.4. Usefulness of Service Platform Concept in Conceptualizing the Mental Health System (Q4)

Question 4 asked respondents if the service platform approach was useful for conceptualizing the mental health system and its underlying segments. Responses were arranged according to two categories: tacit usefulness and conditional usefulness. One respondent did not understand, while another respondent did not want to comment on the concept’s utility without further information. Of the seven remaining respondents, two agreed (explicitly or tacitly) that the concept was useful, while five considered the concept to be useful, subject to conditions.

Two respondents tacitly demonstrated the usefulness of the service platform approach by referencing comparative frameworks (e.g., ‘service elements’ in the NMHSPF or ‘Models of Service’):

*“Well, we certainly aspire to see a number of related service elements/interventions grouped concurrently and/or sequentially, so that we make adequate and complementary responses that address all the life domains relevant to the person with that illness. With severe low prevalence mental illness that is a lot of service elements.”*

*[Respondent 1]* 

*“Service platforms will group services in a similar fashion to our Models of Service. It should group on target group, staffing capability, interventions offered and expected deliverables”*

*[Respondent 3]* 

Even though they both agreed, these two respondents offered contrasting interpretations of the service platform concept. Respondent 1 appeared to be relating the service platform definition to the idea of ‘continuity of care’ and ‘effective coverage’ of the population. This could be viewed as a person- or consumer-centered approach to health care planning. In contrast, the second respondent provided a more mechanistic interpretation by referencing the different sub-units that would be useful for planning activities.

Five respondents considered the service platform concept useful, subject to conditions. For instance, one respondent stated that the usefulness of the approach would depend on how resource allocation is determined and the complexity of the service platform taxonomy:

*“I think the utility depends on how resource allocation is determined. […] If the platform included too many subunits, it would lose utility.”*

*[Respondent 2]* 

One respondent stated that the service platform concept could be useful if it matched services to the health care needs of consumers. They cautioned that health care planning frameworks should not lose sight of the ‘person’:

*“[Yes] if the definition of a Service Platform for mental health includes reference to the people we are trying to service. Without such a reference there is a risk that the individual with the disorder will be lost in the higher level focus of the definition. […] the definition makes no reference at all to the consumers and their disorders.”*

*[Respondent 5]* 

One respondent explicitly refuted the usefulness of the concept:

*“No, as currently defined, it is more applicable to defining an element or a cluster of the elements of the mental health system rather than the system itself. In this context I think it has limitations currently, in particular the words ‘component of’ a continuum of care. There is merit in considering the definition of a service platform in the context of a definition of the mental health system.”*

*[Respondent 4]* 

This comment appeared to refute attempts to encompass the entirety of the mental health system in terms of service platforms. In light of this, we suggest that Respondent 4 found the service platform approach conditionally useful.

#### 3.2.5. Usefulness of Service Platform Concept in Planning and Funding (Q5)

Question 5 asked PAP participants if the service platform approach was useful for planning and funding purposes. Out of seven responses, three explicitly agreed that the concept was useful for the purposes of funding and planning while four considered the concept useful, subject to certain conditions.

One respondent expressed agreement with the concept’s utility and described how the service platform concept could be used to match services to the mental health needs of the population:

*“It is a helpful delineation. If expected Service Platform utilization is understood from population size and demography existing capacity can be mapped, the gap understood and then planning for staged growth, addressing areas of greatest need first can be progressed.”*

*[Respondent 3]* 

The respondent regarded the service platform concept to be a particularly useful tool if it could be used to plan the allocation of resources in relation to demographic data.

Four respondents considered the concept useful, subject to conditions. Many of these respondents reiterated remarks provided in the previous question. One of the respondents recognized the usefulness of the service platform approach for mapping both the ideal and current service system, if it were linked to measurable quantities:

*“Yes, it will assist in delineating elements of the service system and understanding a system, current or ideal. To be of assistance for planning and funding, it will require to [sic] move beyond definitions and refined to include values.”*

*[Respondent 4]* 

Respondent 9 suggested that the service platform definition could be useful for the purposes of planning if it was linked to the attainment of measurable outcomes. This could help service planners gauge the efficiency and/or effectiveness of mental health services:

*“Linked to an outcome analysis, this kind of definition may help us to decide what kinds of service platforms, or resource types, are most efficiently and effectively applied to achieve certain outcomes.”*

*[Respondent 9]* 

Respondent 2 offered insight on the usefulness of the service platform approach in relation to a similar planning framework:

*“[A related framework] set out to define each component of an area based mental health service, including those elements that should be in each area, and those that would be regional or state-wide specialist. I think [the framework] worked well when funding was relatively high. With progressive reduction in funding in real terms, these component parts have been lost or rolled into a more integrated function—e.g., primary mental health, acute assessment, continuing care, etc. So I think to make the platform concept of use also requires consideration of the population covered and the overall resourcing.”*

*[Respondent 2]* 

Several suggestions are offered in the statement above. For instance, the respondent recommends that population groups and the available budget should be taken into consideration when applying the service platform concept. The respondent also mentions that the service system can be considered at different levels of aggregation, apart from platforms. Lastly, geography is acknowledged as an important consideration for planning and funding activities.

Overall, the responses on usefulness echoed the preceding responses on acceptability and comprehensibility. Most respondents stated that the service platform concept could be useful for the purposes of planning and funding, provided that it made explicit reference to other important factors for mental health planning (e.g., population needs, consumer outcomes, overall resourcing, *etc*.). Furthermore, we observed respondents actively deliberating how they could apply the service platform definition in nuanced ways. This implicitly supported the usefulness of the definition.

#### 3.2.6. Suggested Changes to the Service Platform Definition

Only one explicit recommendation was made to modify the wording of the service platform definition (see Respondent 4’s answer to Question 4). In this case, the respondent suggested rewording the phrase ‘component of’ a continuum of care. Apart from this, we identified a number of synonyms and alternative terminology used by respondents when referring to the service platform concept. These alternative words and phrases have been collated in Table 3. Terminology from the NMHSPF was periodically referenced by several respondents.

**Table 3:** List of synonyms and alternative terminology related to the ‘service platform’ definition.

Respondent	Alternative Terminology Used by Respondent
1	Respondent initially understood service platform in terms of ‘packages’ (*cf*. ‘care packages’ used in NMHSPF). Also referred to ‘service elements’ and ‘interventions grouped concurrently and/or sequentially’.
2	Referenced terminology related to the hierarchical taxonomy of services produced by the NMHSPF—*i.e.*, ‘service group’, ‘service stream’, ‘service category’, ‘service element’, ‘service activity’.
3	Related the service platform concept and the way it grouped services to a ‘Models of Service’ concept.
4	Understood a service platform to be a ‘cluster of the elements in the mental health system’.
7	Respondent used the terms ‘service clusters’ or ‘service constellations’ to refer to groupings of services in the mental health system.

Note: NMHSPF—National Mental Health Service Planning Framework.

## 4. Discussion

### 4.1. Discussion of Findings

On balance, we found that most respondents believed the service platform concept to be acceptable, comprehensible and useful—albeit with some caveats. A handful of respondents pointed out terminological issues in the definition that needed to be addressed. The majority of respondents’ feedback highlighted the importance of a range of factors in relation to a service platforms approach to planning. For instance, a number of respondents stated that the service platform approach would be useful for modelling an optimal or ‘ideal’ service system, but may have restricted applicability to the ‘actual’ system.

We intentionally presented the service platform definition devoid of examples to provoke a nuanced investigation of respondents’ views. The majority of respondents articulated some form of self-reported understanding of the service platform concept. The examples participants gave tended to be in the form of an aggregated service system entity coupled with a defined function—which is consistent with the underlying intent of the service platform approach. We also observed that policy makers thought of service entities at a range of levels of aggregation. For some, an aggregation of services could entail a hospital ward, an organization, acute inpatient services, or a grouping of all bed-based services across an entire system. We hypothesize that the level of aggregation might vary depending on what parts of the mental health system the policy maker in question had oversight of. For example, a State government bureaucrat who has oversight of the public hospital system may think at a less aggregated level of services than a Federal government bureaucrat who has oversight of the broader health system, including primary care and population health. The blinded analysis of transcripts prevented us from evaluating responses in light of the position or influence of a respondent.

Respondents considered the service platform definition useful for conceptualizing the mental health system and as a tool for planning and funding decisions, subject to several conditions. One theme that emerged from the analysis of transcripts was the need to ensure that a focus on the consumer was not lost if service platforms were taken up as an organizing principle for resource planning and allocation. Respondents maintained that the service platform definition would not be acceptable or useful to decision makers unless it made explicit reference to the health care needs of the population. Some respondents discussed the evaluation of consumer outcomes with respect to service provision. One respondent even made a direct suggestion to conduct an outcome analysis to determine which service platforms can most effectively and efficiently improve health outcomes. Finally, several respondents acknowledged the highly aggregated nature of the service platform concept and pointed out that it will need to be divided into further sub-components to be useful for mental health planning. However, the utility of this approach could be impaired if it resulted in an overly complex taxonomy of platforms and underlying sub-units.

### 4.2. Moving forward with the Development of the Service Platform Concept

To further develop the service platform concept, we first need to address issues raised by respondents in relation to the terminology of the service platform definition. The word ‘continuum’ turned out to be an ambiguous term that had connotations of an ideal mental health system whereby an individual consumer experienced a ‘best practice’ clinical care pathway or ‘continuity of care’. While the current definition could be framed in terms of a continuum of care, it was not our intention to exclusively limit the concept to these terms. We included the phrase ‘continuum of care’ as a way of mapping service platforms according to the different segments of the mental health intervention spectrum [28,29]. A crude way of picturing this would be to think of a ‘prevention platform’ or a ‘treatment platform’ which would group comparable services that have a distinct role or function along the intervention spectrum. Mental health services could thus be mapped to each discrete cluster of services without reference to a continuum of care. Alternatively, services could also be mapped along a continuum of care as the range of platforms reflects the entire intervention spectrum. Based on these remarks, we have considered changing the wording of the definition to, ‘A service platform is a grouping of related services that are similar in resource type and constitute a component within a spectrum of care’. Consideration is also being given to a less ambiguous term than ‘component’, such as ‘part’ or ‘element’. The next phase of our project will test these modifications.

A handful of respondents stated that the service platform concept would be useful for modelling an ideal, rather than an actual, mental health service system. In some respects, we are indifferent towards the status quo (*i.e.*, the current system)—which is the culmination of a historically ad hoc, crisis-driven mental health reform process. We maintain that the raison d’etre of this study is to provide mental health policy makers and service planners with a visionary concept that can supplant previous models of reform and provide guidance towards realizing an optimal mental health service system. All in all, we found it reassuring that respondents found the service platform concept useful for describing an ideal service system. Even so, we would also like the definition to be useful for mapping the current service system and to highlight gaps between the current and ideal service systems. In light of these participants’ remarks, it appears that the current definition will require further refinement—possibly using a set of guiding principles attached to the definition that clarify how current services relate to the service platform concept.

The development and testing of the service platform definition was the first step in the process to develop a useful tool for mental health decision making. The next step in our program of work is to identify and define the service platforms and the underlying sub-components that reflect the current and ideal mental health system in Australia. Given the complexities of partitioning the mental health service system, it follows that these service platforms will need to be refined, tested and further explained in a separate publication. To this end, we are planning to formulate and test a set of guidelines to determine how service platforms and their underlying branches of services should be partitioned (*cf*. the ‘service tree’ produced by the European Service Mapping Schedule). The ‘principle of parsimony’ will be an important consideration to ensure that platforms are not subdivided any more than is necessary for the purposes of planning and funding.

**Figure 1:**
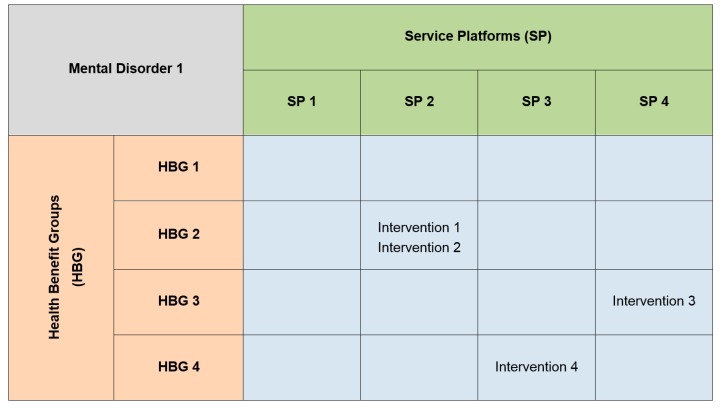
Mock example of a Health Benefit Group/Service Platform Matrix.

The service platform concept underpins a service system taxonomy and is not explicitly aimed at accounting for the needs of consumers or the outcomes they seek from mental health services. We will address recommendations to reference population needs and service outcomes by integrating the service platform idea with a concept called the ‘Health Benefit Group’ (HBG) [30,31,32]. The HBG is a complementary service modelling construct to the Health Resource Group, which classifies groupings of individuals, ‘who have similar health and wellbeing needs and who can be expected to achieve similar outcomes given similar interventions’ [32]. Tabulating a two-dimensional matrix of HBGs and SPs is a way of relating service provision to the health care needs of the population. Figure 1 shows a mock example of a HBG/SP classification matrix that tabulates rows of HBGs to columns of SPs. The intersecting cells between each HBG row and SP column can be a useful way of visualizing how resources are allocated in a system to fund services / address population needs. Intersecting cells can be filled with relevant information on the numbers of people who receive services based on their health care needs, in addition to the type and quantity of services provided in the current system (e.g., HBG2/SP2, HBG3/SP4 and HBG4/SP3). Priority setting information on disorder-level interventions could then be applied by substituting the current provision of interventions with their cost-effective alternatives.

The link between the service platform concept and population health needs can be demonstrated by considering an example of a possible platform, for example ‘bed-based mental health care’. This platform would comprise services that provide clinical care necessitating an overnight stay in a specialized mental health care facility, for example, people requiring management of the symptoms of an acute episode of illness, and people with disability-related needs who require ongoing monitoring and care at a community residential service.

In addition, the classification matrix could be used to map the provision of services for a mental disorder such as major depressive disorder or schizophrenia. If matrices were collated for multiple disorders, then it would be possible to aggregate them into a single HBG/SP classification matrix that represents all the services/interventions delivered by the broader system. A service system framework centered on the HBG/SP classification matrix can thus be used to map mental health services to different mental health needs for both the current and ideal service systems, and to measure the gap between the two. However, the list of SPs and HBGs needs to be refined to ensure alignment with the mental health system. 

We are ultimately planning to use the HBG/SP matrix to design three hypothetical models of the mental health service system in Australia. The first model (Model A) seeks to estimate the burden averted by the current system of platforms, based on the existing allocation of resources and the effective coverage of services. Next we will estimate the burden able to be averted by each service platform (and therefore the system as a whole) under the scenario that each platform was delivering the most effective and cost-effective interventions with the current allocation of resources (Model B). Finally, we could estimate the maximum reduction in disease burden with an optimal allocation of additional resources, if each service platform was delivering effective and cost-effective interventions (Model C). These models can provide guidance to mental health policy makers on how they should allocate current or additional resources between platforms to achieve optimal outcomes for service consumers. In addition to these hypothetical scenarios, the service platform approach could be a useful framework for comparing the theoretical efficacy of interventions provided by each platform relative to the actual effectiveness of these interventions on the ground.

### 4.3. Strengths and Limitations

The main strength of our study is that it aims to test the usefulness of concepts used in health systems modelling with the end-users of modelling, prior to model development—*i.e.*, with Australian policy makers who have direct experience in planning and funding decisions within a different jurisdiction of the Australian mental health system. Several protocols were established to reduce the risk of bias when analyzing questionnaire transcripts (e.g., our decision to blind the analysis of respondent transcripts and foreshadowing strategies for assessing disagreement).

Conducting a blind analysis of transcripts is both a strength and a limitation. While it may reduce the risk of analytical bias by facilitating the equal weighting of responses, there is also a risk that such an analysis can lead to spurious results. For example, it could be argued that different respondents’ views should be weighted differently according to their level of influence on policy development.

One notable limitation involved our decision to provide a restricted explanation of the service platform concept to respondents. We had decided to limit the extent to which we explained the concept to avoid artificially inducing biased responses that have been prompted by the disclosure of excessive background information. We also sought to test whether the definition was sufficient in itself to provide a clear understanding of what is meant by the term ‘service platform’. Despite this, the lack of additional explanation could have resulted in respondents having an unclear understanding of the actual concept being tested and possibly led to an artefact in the qualitative findings.

There is a risk that our sample was too small for our qualitative analysis to reach thematic saturation. However, we note that the target population for this research is very small. One final limitation is that the findings of this study may not be generalizable beyond high-income country settings like Australia. Further studies will need to be conducted to validate the utility of the service platform approach to policy makers in an international context.

### 4.4. Future Research Directions

This study used an approach that sought to directly consult with the end-users of our research (*i.e.*, policy makers) with the aim of designing implementable priority setting models and is intended to be the first in an ongoing process of stakeholder engagement. This varies from previous approaches to research translation insofar as stakeholder views are incorporated into model development during the formative stages, rather than elicited afterwards to facilitate implementation. However, systematic mental health reform and the implementation of an optimal service system will be constrained by shifting socio-political and legal factors and frameworks. Thus, a degree of advocacy in advancing the end result is inescapable and it is imperative that any priority setting model constructed using a service platform approach is complemented by a communication strategy.

## 5. Conclusions

We have developed a service platform definition and tested it with a group of mental health policymakers. Our findings suggest that the service platform concept could be a useful way of aggregating mental health services as a means for presenting evidence to policy makers to inform priority setting. Further development and testing of the concept is required.

## Supplementary Files

Supplementary File 1
