# Supplementary material for: Developing a Service Platform Definition to Promote Evidence-Based Planning and Funding of the Mental Health Service System
Source: Int J Environ Res Public Health. 2014 Nov 26;11(12):12261–82. doi: 10.3390/ijerph111212261 (PMC4276613; doi:10.3390/ijerph111212261)
Supplement: Supplementary File 1 [file ijerph-11-12261-s001.pdf]

Service Platforms

Defining an Organisational Framework to *Facilitate the Reform of Mental Health Services in Australia*

A survey for the NHMRC Centre of Research Excellence in Mental Health Systems Improvement (CREMSI)

http://mhsystems.org.au/

Complete the questions on the following pages by entering your responses in the blank text fields provided. Please save your answers after completing the survey and email them to: c.meurk@qcmhr.uq.edu.au

The University of Queensland
Australia

Background Information

It is important that mental health policy makers and service system planners have good quality information on how to allocate existing budgets to improve mental health outcomes.

There is research capacity in Australia to design an optimal mental health system. However, evidence produced by researchers is often presented in a way that is not compatible with the decision making frameworks used by mental health policy makers.

Health service planners are regularly tasked with allocating resources across broad entities of the mental health system (e.g., primary care or bed-based hospital services). It follows that research evidence, from relevant disciplines such as health economics and service system planning, should be presented in a way that reflects this type of decision making.

Researchers, particularly health economists, conventionally present evidence according to the types of intervention that should be funded to address different mental disorders in the population (e.g., SSRI's for depression or internet-based CBT for anxiety). However, evidence useful to planners should be presented in terms of the broad categories of allocation used in health care budgets across an entire system.

Researchers and policy makers need to jointly develop a novel analytic framework that will lead to the generation of research evidence which can be used to allocate resources in the mental health system.

~~~

We have developed a 'Service Platform' concept based on our knowledge of the policy making process. We have found that this concept is commonly used by policy makers and may be useful when generating and presenting economic evidence on the allocation of mental health resources. However, no precise definition of this concept currently exists in the published literature.

In this survey, we wish to test a definition we have developed. We would like to find out your views of this definition and to ascertain whether you think the concept is useful for the purposes of planning mental health care services. Please note that there are no right or wrong answers – we are simply interested in obtaining your perspective on the matter.

NB: The answer boxes will automatically expand as you type.
~~~

Questionnaire

One definition of a service platform is:

> *"A service platform is a grouping of related services that are similar in resource type and constitute a component of a continuum of care."*

~~~

We would like to ask you the following questions in relation to the service platform definition:

1. Can you tell us if this definition of a Service Platform is consistent with how you think the health system is organised? Why or why not?

   Click here to enter text.

2. Does this concept make sense to you? Why or why not?

   Click here to enter text.

3. Can you provide us with examples of Service Platforms?

   Click here to enter text.

4. Do you think this definition of a Service Platform is a useful way to conceptualise the mental health system? Why or why not?

   Click here to enter text.

5. Do you think this definition of a Service Platform delineates different types of services in a way that is helpful for planning and funding?

   Click here to enter text.

6. Is there anything else about the Service Platform concept that you would like to share with us?

   Click here to enter text.
~~~

Thank you!

Thank you for taking the time to complete and submit this survey. Your insight and information will be valuable to us as we endeavour to produce better quality evidence for mental health system reform.

Before submitting this survey, do you have any thoughts about this exercise (or the PAP process) that you would like to share with us?

Click here to enter text.

Please email your completed survey by clicking the button provided below, or by attaching it to an email addressed to: c.meurk@qcmhr.uq.edu.au
